# Is sleep the new treatment for pain? Two issues need resolving before deciding

**DOI:** 10.1093/sleep/zsae089

**Published:** 2024-04-18

**Authors:** David M Klyne, Michelle Hall

**Affiliations:** NHMRC Centre of Clinical Research Excellence in Spinal Pain, Injury and Health, School of Health and Rehabilitation Sciences, The University of Queensland, Brisbane, QLD, Australia; Sydney Musculoskeletal Health, The Kolling Institute, School of Health Sciences, University of Sydney, Sydney, NSW, Australia

It is well known that chronic pain [1, 2] and poor sleep [3–5] are enormous and growing health problems. Less recognized, however, is the relationship between the two. Poor sleep cooccurs with chronic pain in up to 90% of cases and is associated with pain severity [6–9]. Contrary to the traditional view that pain interferes with sleep, emerging studies including our own preclinical work, suggest the opposite—poor sleep increases pain [6, 7, 10–12]. Moreover, our group has shown that poor sleep predicts a clinically relevant increase in next-day low back pain symptoms (i.e. “flare”) [13] and, when in conjunction with specific immune profiles, the transition from acute to chronic pain [14]. This body of work opens the enticing, yet under-investigated, possibility that interventions aimed at sleep could improve and perhaps even prevent chronic pain. The potential implications are huge as chronic pain and poor sleep on average affect more than 20% [1, 2] and 35% [4, 15] of the world’s population at any one point in time, respectively. However, two issues need resolving before sleep can be confirmed as a legitimate target for preventing and managing chronic pain.

First, mechanisms linking sleep and pain are diverse and unclear. One likely explanation involves the capacity for poor sleep to drive inflammatory mechanisms that sensitize pain pathways [16]. Sleep is a powerful modulator of systemic inflammation [17, 18]. The different stages of sleep (i.e. rapid eye movement and the three stages of non-rapid eye movement) differently regulate nocturnal levels of inflammatory factors (e.g. cytokines), and this lays the foundation for inflammatory levels throughout the day [19–22]. Sleep loss or disturbance can alter this immune balance via activating the “stress response” (i.e. hypothalamic-pituitary-adrenal axis and sympathetic nervous system activation, see [18, 23–25]) and/or inflammatory signaling pathways (e.g. those involving nuclear factor-κB [NF-κB], activation protein-1 [AP-1], and signal transducer and activator of transcription [STAT] family proteins [17, 26–28]). This can result in a transient shift in the temporal pattern of inflammatory responses, with increased levels of pro-inflammatory cytokines during the day rather than during the night [19, 29]. These effects are observed after as little as one night of partial sleep loss [7, 30–32]. Repeated or persistent periods of sleep loss/disturbance can lead to excessive and sustained activation of this inflammatory response, leading to chronically elevated systemic inflammation [33–35]. Many of the cytokines and factors involved in this “inflammatory response” are known to amplify pain via directly sensitizing peripheral and central nervous system tissues [36, 37]. Despite this knowledge, no studies have yet tested and confirmed these “complete” pathways linking sleep and the enhancement or chronification of pain. In fact, only one study has tested whether poor sleep drives the transition from acute to chronic pain. In that study, rats exposed to regular sleep disturbance for 4 weeks after acute limb injury displayed prolonged forepaw sensitivity and enhanced systemic levels of brain-derived neurotrophic factor (BDNF) [11]. The latter point is important because BDNF is involved in sensitizing pain pathways via its strong capacity to regulate synaptic plasticity [38–40]. Interestingly, key pro-inflammatory factors involved in both sleep and pain (e.g. tumor necrosis factor-α and interleukin-6) are also known triggers for BDNF release [40, 41], highlighting a potential crucial mechanism by which sleep-induced inflammation indirectly (in addition to directly, e.g. via cytokines acting on nociceptive neurons) acts on the nervous system to increase and maintain pain. Confirmation of these sleep-activated pathways will require disentangling the role of inflammation in these processes at the systemic, tissue, and molecular level as pain evolves from acute to chronic. Equally important will be to establish whether *improved* sleep impacts pain via reverse actions on similar pathways.

Second, a major barrier to realizing the true impact of sleep on pain is that methods for accurately quantifying sleep “quality” are impractical for long-term personal/home use and require extensive equipment that limits comfort and movement, and ultimately sleep quality [42–44]. For example, the gold standard for objectively monitoring sleep quality is polysomnography (PSG) which involves the costly combined recordings of brain activity, muscle tone, heart activity, respiratory rate, nasal airflow, and eye movement, among other physiological features [45, 46]. As a result, “real-world” studies have traditionally relied on self-reported questionnaires that invariably capture *subjective* aspects of sleep quality (e.g. sleep efficiency, latency, and disturbance), but are limited by recall bias and cannot accurately, or at all, inform sleep architecture (i.e. amount of the different sleep stages) and the amount, duration and frequency of awakenings (i.e. sleep fragmentation) across a sleep episode. Consumer wearable technologies such as wrist-worn activity monitors have been pegged as the solution to overcoming these barriers. These devices have traditionally relied on accelerometer data to estimate basic sleep versus wake parameters with questionable precision [47]. However, newer generation devices contain other sensors for monitoring physiological signals (e.g. heart rate, heart rate variability, blood oxygen saturation, and respiration rate) that are used as additional inputs into proprietary sleep algorithms to improve and expand sleep measures to include quality indices such as basic sleep architecture (e.g. percent time spent in non-rapid *vs.* rapid eye movement sleep) [48–52]. Although promising, these “quality” measures of sleep have not yet been robustly validated (i.e. against PSG setups) for clinical or research use in real-world settings or/in pain populations. Addressing this issue will be critical for opening the door to large-scale home-based sleep studies that can confirm the role of sleep in pain and testing sleep interventions.

Sleep as a first-line treatment for pain is within sight. Confirmation of mechanisms of sleep-induced changes in pain would likely guide clinical advice and self-management strategies for pain relief, and provide an evidence-based basis for developing, refining, and testing sleep interventions for improved prevention and management of chronic pain. One “ready-to-test” sleep intervention that has been shown to concurrently improve sleep and pain is cognitive behavioral therapy for insomnia (CBT-I) [53–58]. A major advantage of CBT-I is its potential to benefit a substantial proportion of pain suffers given insomnia is the most common sleep disorder [59] and 50% of those with insomnia experience chronic pain [60, 61]. Sleep interventions may also have an additive effect on pain relief when combined with core first-line treatments such as exercise, which is also thought to contribute to pain relief by reducing inflammation [62, 63]. Closely related to this line of inquiry is our preclinical work showing exercise protects against the negative effects of poor sleep on pain and the transition to chronicity [11]. These observations are particularly relevant for subgroups of pain suffers that cannot easily modify their sleep (e.g. due to shiftwork constraints). The successful validation of simple wearables to accurately monitor neurological sleep processes will lay the foundation to test and confirm these ideas in the real-world.

## Data Availability

Data from studies that are mentioned and owned by either Drs. Klyne or Hall will be made available following a written request to the corresponding author, along with a summary of the planned research.
